# Wildlife Trade and Global Disease Emergence

**DOI:** 10.3201/eid1107.050194

**Published:** 2005-07

**Authors:** William B. Karesh, Robert A. Cook, Elizabeth L. Bennett, James Newcomb

**Affiliations:** * Wildlife Conservation Society, Bronx, New York, USA;; † Bio-Economic Research Associates, Boulder, Colorado, USA

**Keywords:** Hepatitis, HIV, SARS, tuberculosis, healthcare worker, occupational safety, vaccinia

## Abstract

The global trade in wildlife provides disease transmission mechanisms that not only cause human disease outbreaks but also threaten livestock, international trade, rural livelihoods, native wildlife populations, and the health of ecosystems. Outbreaks resulting from wildlife trade have caused hundreds of billions of dollars of economic damage globally. Rather than attempting to eradicate pathogens or the wild species that may harbor them, a practical approach would include decreasing the contact rate among species, including humans, at the interface created by the wildlife trade. Since wildlife marketing functions as a system of scale-free networks with major hubs, these points provide control opportunities to maximize the effects of regulatory efforts.

Threats to global health and risk factors for emerging infectious diseases run the gamut from climate change to poverty to security issues, but few are as immediately manageable as the global trade in wildlife. Trade in wildlife provides disease transmission mechanisms at levels that not only cause human disease outbreaks but also threaten livestock, international trade, rural livelihoods, native wildlife populations, and the health of ecosystems. Quantifying the global wildlife trade is almost impossible since it ranges in scale from local barter to major international routes, and much is conducted illegally or through informal networks. Some estimates indicate that ≈40,000 live primates, 4 million live birds, 640,000 live reptiles, and 350 million live tropical fish are traded globally each year (1). Live wildlife in markets in Guangzhou, China, trade in masked palm civets, ferret badgers, barking deer, wild boars, hedgehogs, foxes, squirrels, bamboo rats, gerbils, various species of snakes, and endangered leopard cats, along with domestic dogs, cats, and rabbits (2). After the outbreak of severe acute respiratory syndrome (SARS) in 2003, 838,500 wild animals were reportedly confiscated from the markets in Guangzhou (3). Wild mammals, birds, and reptiles flow daily through trading centers, where they are in contact with persons and with dozens of other species before they are shipped to other markets, sold locally, or even freed and sent back into the wild as part of religious customs such as merit release (4) or because they become unwanted pets. In a single market in North Sulawesi, Indonesia, up to 90,000 mammals are sold per year (5). In a survey conducted at 1 market in Thailand for 25 weekends, >70,000 birds, representing of 276 species, were sold (6). A similar survey of 4 markets in Bangkok in 2001 found that of 36,537 observed birds; only 37% were native to Thailand, while 63% were nonnative species (7).

In lieu of precise trade data, we conservatively estimated that in East and Southeast Asia, tens of millions of wild animals are shipped each year regionally and from around the world for food or use in traditional medicine. The estimate for trade and local and regional consumption of wild animal meat in Central Africa alone is >1 billion kg per year (8), and estimates for consumption in the Amazon Basin range from 67 to 164 million kilograms annually (9,10); for mammals alone, this consumption consists of 6.4 million to 15.8 million individual animals (11). In Central Africa, estimates of the number of animals consumed by humans annually vary, but 579 million has been proposed (12).

Hunters, middle marketers, and consumers experience some type of contact as each animal is traded. Other wildlife in the trade is temporarily exposed, and domestic animals and wild scavengers in villages and market areas consume the remnants and wastes from the traded and potentially traded wildlife. These numbers combined suggest that at least some multiple of 1 billion direct and indirect contacts among wildlife, humans, and domestic animals result from the wildlife trade annually. The increasingly global scope of this trade, coupled with rapid modern transportation and the fact that markets serve as network hubs rather than as product endpoints, dramatically increases the movement and potential cross-species transmission of the infectious agents that every animal naturally hosts.

Since 1980, >35 new infectious diseases have emerged in humans (13), ≈1 every 8 months. The origin of HIV is likely linked to human consumption of nonhuman primates (14). Recent Ebola hemorrhagic fever outbreaks in humans have been traced to index patient contact with infected great apes that are hunted for food (15). SARS-associated coronavirus has been associated with the international trade in small carnivores (16), and a study comparing antibody evidence of exposure to this coronavirus demonstrated a dramatic rise from low or zero prevalence of civets at farms to an approximately 80% prevalence in civets tested in markets (17).

The inadvertent movement of infectious agents due to the wildlife trade is not limited to human pathogens but also affects pathogens of domestic animals and native wildlife. H5N1 type A influenza virus was recently isolated from 2 mountain hawk eagles illegally imported to Belgium from Thailand (18). A paramyxovirus highly pathogenic for domestic poultry entered Italy through a shipment of parrots, lovebirds, and finches imported from Pakistan for the pet trade (19). Monkeypox was introduced to a native rodent species and subsequently to humans in the United States by importing wild African rodents from Ghana for the US pet trade (20). Chytridiomycosis, a fungal disease now identified as a major cause of the extinction of 30% of amphibian species worldwide, has been spread by the international trade in African clawed frogs (21). Merit release of wild birds and reptiles that have passed through markets provides another avenue for introducing novel infectious agents into the wild (4) and warrants further attention (Figure).

**Figure:**
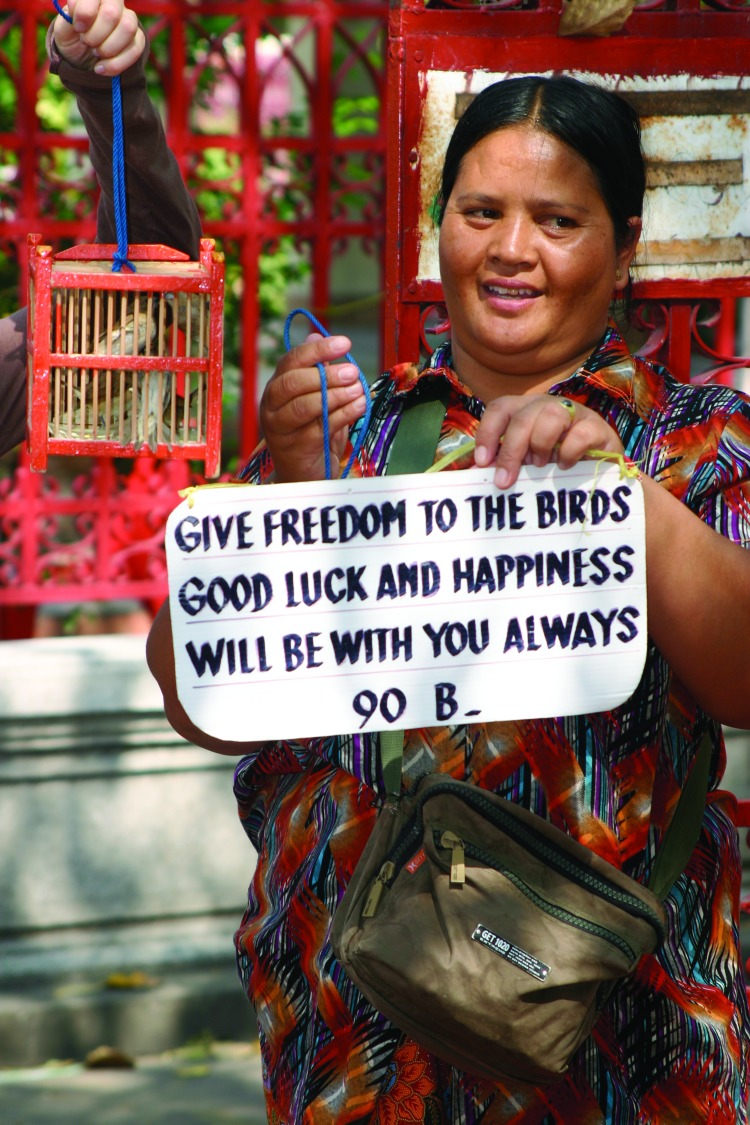
Vendor selling wild-caught birds for release at a religious shrine in Thailand. (Photo by W.B. Karesh.)

Many diseases are transmitted through the same species of parasites carried by imported animals. For example, from November 1994 to January 1995, US Department of Agriculture personnel inspected 349 reptile shipments from 22 countries containing 117,690 animals. Ticks were removed from animals in 97 shipments, and infested shipments included 54,376 animals (22). Ticks carry many diseases that threaten livestock and human health, including heartwater disease, Lyme disease, and babesiosis.

The possibility of emerging infectious diseases spreading between persons and animals is rising, fueled by human activities ranging from the handling of bushmeat and the trade in exotic animals to the destruction or disturbance of wild habitat (23–25). In a list of 1,415 human pathogens, 61% are known to be zoonotic, and multiple host pathogens are twice as likely to be associated with an emerging infectious disease of humans (26). Seventy seven percent of pathogens found in livestock are shared with other host species (27).

In addition to the direct health effects of the pathogens on persons and animals, animal-related disease outbreaks have caused hundreds of billions of dollars of economic damage globally, destabilizing trade and producing devastating effects on human livelihoods. The rash of emerging or reemerging livestock disease outbreaks around the world since the mid 1990s, including bovine spongiform encephalopathy, foot-and-mouth disease, avian influenza, swine fever, and other diseases, has cost the world's economies $80 billion (28). In early 2003, the United Nation's Food and Agriculture Organization reported that more than one third of the global meat trade was embargoed as a result of mad cow disease, avian influenza, and other livestock disease outbreaks. Efforts to control the spread of avian influenza in Asian countries since 2003 have required the culling of >140 million chickens (29). The projected growth of industrial livestock production in nonindustrialized countries to meet global protein demand will increase the impact of future disease outbreaks on economic and food supply security. Some of these outbreaks will inevitably be linked to the trade in wildlife.

Rather than attempting to eradicate pathogens or the wild species that may harbor them, a practical approach to decrease the risk for the spread of infectious diseases would include decreasing contact among species. Closing down retail poultry markets in Hong Kong for 1 day per month reduced the rate of H9N2 avian influenza virus in market birds (30). Little equivalent research has been conducted in market systems that sell wildlife, but an analogous approach to the precautionary principle (31) would be an appropriate action to take before the next outbreak or pandemic. Since wildlife markets are a system of networks with major hubs, these trading points provide practical control opportunities to maximize the effects of regulatory efforts (32). Focusing efforts at markets to regulate, reduce, or in some cases, eliminate the trade in wildlife could provide a cost-effective approach to decrease the risks for disease for humans, domestic animals, wildlife, and ecosystems.
